# Differences in dietary pattern by maternal age in the Born in Bradford cohort: A comparative analysis

**DOI:** 10.1371/journal.pone.0208879

**Published:** 2018-12-13

**Authors:** Katie Marvin-Dowle, Karen Kilner, Victoria Burley, Hora Soltani

**Affiliations:** 1 Centre for Health and Social Care Research, Sheffield Hallam University, Sheffield, United Kingdom; 2 School of Food Sciences and Nutrition, University of Leeds, Leeds, United Kingdom; Universita degli Studi di Milano, ITALY

## Abstract

**Objective:**

Explore associations between dietary patterns and maternal age

**Design:**

Population based cohort study

**Setting:**

Maternity department of a large hospital in northern England

**Sample:**

Women delivering a singleton at Bradford Royal Infirmary between March 2007 and December 2010 (N = 5,083).

**Methods:**

Survey data including maternal dietary patterns derived from food frequency questionnaire data using principal component analysis (PCA) were compared by maternal age using one-way ANOVA and chi-squared as appropriate.

**Main outcome measures:**

Dietary pattern PCA scores, supplement use, familiarity and compliance with 5-a-day fruit and vegetable recommendations, consumption of cola, maternal BMI.

**Results:**

Three distinct dietary patterns were derived from the data; snack and processed foods, meat and fish and grains and starches. Mean PCA score for snack and processed foods was higher among women aged ≤19 (0.6, CI 0.4 to 0.8) than women aged 20–34 (-0.02, CI -0.1 to 0.01) and those aged 35≥ (-0.3, CI -0.4 to -0.2). Women aged 35≥ had a significantly higher mean PCA score for the grains and starches dietary pattern (0.1, CI 0.03 to 0.3) compared to both the 20–34 years (-0.01, CI -0.05 to 0.02) and the ≤19 (-0.04, CI -0.2 to 0.1) groups. No differences were observed between groups in mean PCA scores for the meat and fish dietary pattern. Adolescent women also had higher intakes of sugar sweetened cola (0.9 cups per day, CI 0.7 to 1.1) and reported lower levels of fruit and vegetable and supplement intake. Women aged 35≥ had a higher mean BMI (28.0, CI 27.5 to 28.4) and higher prevalence of overweight (36.8%) and obesity (29.6%, p<0.001).

**Conclusions:**

Significant differences were observed between age groups both in terms of diet quality and BMI. Interventions targeted by age group may be advantageous in improving maternal nutrition and contribute to healthy pregnancies.

## Introduction

Nutrition during pregnancy is a well-established modifiable factor which has the potential to impact upon the health and well-being of both mother and child[1]. Study of nutritional intake and nutrient sufficiency in populations is difficult owing to the fact that individuals will have differing nutritional needs due to differences in environmental and genetic factors such as energy expenditure, body composition and metabolism. This is particularly true when investigating the relationship between nutrition and maternal and neonatal health as this is complicated by other biological, demographic and social factors, which vary substantially between different populations.

One such factor is the age of the mother during pregnancy. Adolescence is a time of substantial physiological change during which the nutritional needs of adolescent women are likely to differ from those of older women. Previous work examining the impact of maternal growth on outcomes in adolescent pregnancies suggested that nutrient partitioning between mother and child in growing adolescents negatively affected foetal growth and prematurity[2]. A further study[3] also found mean birthweight in babies born to adolescent women to be lower than those born to adult women; however this was not related to maternal growth in the adolescent participants. This suggests that the relationship between maternal age, maternal growth and foetal growth is complex and warrants further investigation, given that low birthweights are associated with short and long term adverse health outcomes[4].

A recent systematic review of nutritional status of pregnant adolescents in developed countries found that intakes of energy, fibre and a number of micronutrients were below recommended levels in this population[5]. The study also found that there was some cause for concern with regard to biological markers of iron and selenium status.

Similar results were however reported in two reviews which were not confined to adolescent populations[6–7]. This suggests that while there is evidence that diet quality may vary by maternal age, more work to examine the nature of these differences would be advantageous.

These previous works suggest that there may be nutritional issues that may impact on the health of both mother and child which vary by maternal age. This study therefore aims to assess differences in dietary pattern by maternal age in the Born in Bradford cohort.

## Methods

Born in Bradford is a largely bi-ethnic cohort of approximately 13,500 children with high levels of socio-economic deprivation. Pregnant women who were booked to deliver at Bradford Royal Infirmary were invited to join the cohort between March 2007 and December 2010. A detailed profile of the cohort has been published elsewhere[8]. This study uses data collected in phase 2 of the Born in Bradford cohort study[8] to examine dietary patterns of women delivering singletons who took part in the study. The Born in Bradford study is a prospective cohort study for which participants were recruited during pregnancy. All women booked for delivery at Bradford Royal Infirmary are offered an oral glucose tolerance test (OGTT) at 26–28 weeks gestation. Women were invited to participate in the Born in Bradford study when attending this appointment. Informed consent was obtained from all participants women were asked to complete a baseline questionnaire. All participants including those aged under 18 were considered competent to consent on their own behalf. Documents including participant information, consent forms and questionnaires are available on the Born in Bradford website[9] Recruitment took place between March 2007 and December 2010 and over 80% of women eligible in this period agreed to take part. A sub-set of the whole cohort completed a food frequency questionnaire (FFQ) as part of the baseline data collection; therefore data relating to 5,083 pregnancies was available for this analysis. Ethical approval for the study was granted by Bradford Research Ethics Committee (ref no. 07/H1302/112).

### Variables in the analysis

The following demographic characteristics of the sample reported in the baseline questionnaire were explored; ethnicity, marital status, parity, education, smoking during pregnancy, alcohol and illegal drug use during pregnancy and index of multiple deprivation (IMD) score (IMD is the official measure of relative deprivation for small areas in England and combines information from seven domains of deprivation; income, employment, education, health, crime, housing and environment)[10]. Body mass index and number of weeks gestation at the booking appointment (usually before 12 weeks of pregnancy) were obtained from medical records. Variables related to nutrition examined were: use of any vitamins or iron supplements in the last 4 weeks, use of Pregnacare multivitamins at least twice per week, familiarity with 5-a-day fruit and vegetable recommendations, consumption of 5 fruit and vegetable portions per day and sources of advice about healthy eating, number of cups of regular (sugar sweetened) and diet (artificially sweetened) cola consumed per day. Dietary patterns were derived from FFQ data using principal component analysis as described below.

### Statistical analysis

Due to the focus of this study being on detecting differences between age groups the study population was categorised into three groups for analysis according to maternal age; ≤19, 20–34 and 35≥ years, with women aged 20–34 being considered as the reference group. Statistical analysis was undertaken using SPSS 24.

### Principal component analysis

In order to evaluate nutrition in this population a series of statistical analyses were conducted using responses to the FFQ and applying principal component analysis (PCA) to identify distinct patterns of food types consumed. The FFQ was completed at 26–28 weeks gestation and refers to food intake during the preceding 4 weeks. There were a total of 37 items included in the FFQ, details of which are given with the results of the PCA. Since the original objective of the FFQ was to characterise the intakes of key food items only, it was not appropriate to derive an estimate of total energy intake. Responses to the food frequency questionnaire were given on either an eight (1 = Rarely or never, 8 = 5+ times per day) or five (1 = Rarely or never, 5 = 7+ times per week) point scale, these responses were therefore transformed to give an estimation of frequency per day in order to standardise questions asked in different formats.

Individual items on the FFQ were then grouped by type and total frequency of consumption of different food groups estimated. The grouped nutritional variables were then used as the basis for a PCA in order to examine the dietary patterns which exist within the data set. PCA was selected as it allows for a larger number of variables to be reduced and underlying factors exposed.

### Analysis of differences between age groups

Differences between maternal age groups in both the demographic and nutrition related variables were explored using Chi-Square for categorical data and one-way ANOVA for continuous data; categorical variables are presented as percentages and continuous variables as means and 95% confidence intervals. Logistic regression analyses were used to compare the rate of each of the categorical nutrition related variables by age group and differences between groups estimated using odds ratios. Multivariate logistic regression models were then used to adjust these comparisons for confounding variables.

Crude and adjusted odds ratios (OR and aOR) are therefore presented with 95% confidence intervals. Index of multiple deprivation (IMD) score and maternal ethnicity (white British, Pakistani or any other ethnicity) were included as covariates in the adjusted analysis, due to the association of these variables with dietary pattern identified in the literature[11–12]. Where dependent variables were dichotomous, binary logistic regression models were produced. There were also two variables with more than two categorical outcomes for which multinomial logistic regression models were produced. In order to examine the continuous variables regarding consumption of cola linear regression models were produced and dummy variables created to enable the inclusion of categorical independent variables in the model.

In the multivariate regression models for this study there is no clear logical or theoretical basis for assuming any variable to be prior to any other, either in terms of its relevance to the research goal of explaining phenomena, or in terms of a hypothetical causal structure of the data. For this reason a simultaneous method of including independent variables in the multivariate regression models was considered to be most appropriate, as opposed to a stepwise method.

Due to the assumptions required to produce statistical models not being met for the dietary pattern scores (the distribution of the dietary pattern scores is significantly skewed, even following attempts to transform the data) it was not possible to adjust the analysis of these variables for potentially confounding variables.

## Results

### Characteristics of the sample

Data were available for 5,083 pregnancies for this analysis; demographic characteristics of the participants included in the study are shown in Table 1. Most participants in the cohort were aged 20–34 (84%) with 9.5% aged 35 or over and 6.5% aged 19 or under. The sample has a diverse ethnic mix consisting of 46.8% women describing themselves as of Pakistani origin, 38.4% white British and 14.8% of other ethnicities; this distribution of ethnic groups was roughly consistent across the age groups with the exception of the adolescent group which was significantly different. Among women aged 19 and under only 17.6% were of Pakistani ethnicity and 69.4% were white British, the proportion belonging to other ethnic groups was similar to other age groups. There were other significant variations in the characteristics of the sample by maternal age. Women in the adolescent age group were more likely to be unmarried or living with a partner, to be expecting their first child and to have completed lower levels of education compared to older women. Women in the adolescent age groups were also more likely to have smoked or used recreational drugs during pregnancy, however there was no difference in reported alcohol use in the first trimester between age groups, and adolescents were less likely to have used alcohol since the fourth month of their pregnancy compared to older women. Women in the oldest age category were most likely to be overweight or obese while adolescent women were found to have higher prevalence of underweight. IMD score decreased as maternal age increased suggesting adolescent women lived in areas of higher deprivation. Adolescent women also booked with a midwife for antenatal care later than older women.

**Table 1:** Demographic characteristics.

	≤19		20–34		35≥		Total		
	N	%	N	%	N	%	N	%	p =
**Whole Cohort**	330	6.5	4269	84.0	484	9.5	5083	100.0	
**Ethnicity**	** **	** **	** **	** **	** **	** **	** **	** **	
Pakistani	58	17.6	2052	50.0	266	41.9	2376	46.8	<0.001
White British	229	69.4	1454	35.4	264	41.6	1947	38.4	
Any other ethnicity	43	13.0	602	14.7	105	16.5	750	14.8	
**Marital Status**	** **	** **	** **	** **	** **	** **	** **	** **	
Married	48	14.5	2869	69.9	493	77.9	3410	67.3	<0.001
Not married—living with partner	84	25.5	704	17.1	85	13.4	873	17.2	
Single	198	60.0	533	13.0	55	8.7	786	15.5	
**Parity**	** **	** **	** **	** **	** **	** **	** **	** **	** **
0	295	90.2	1571	39.0	112	17.9	1978	39.7	<0.001
1	29	8.9	1296	32.2	164	26.2	1489	29.9	
2	3	0.9	723	18.0	121	19.4	847	17.0	
3 or more	0	0.0	435	10.8	228	36.5	663	13.3	
**Highest level of education**	** **	** **	** **	** **	** **	** **	** **	** **	
Less than 5 GCSEs* grade A-C or equivalent	129	39.1	825	20.0	184	29.0	1138	22.4	<0.001
5 or more GCSEs grade A-C or equivalent	152	46.1	1307	31.7	132	20.8	1591	31.3	
A-levels** or higher	21	6.4	1696	41.2	248	39.1	1965	38.7	
Other/unknown	28	8.5	290	7.0	71	11.2	389	7.7	
**Smoked during pregnancy**	** **	** **	** **	** **	** **	** **	** **	** **	** **
Yes	159	48.2	644	15.7	62	9.8	865	17.1	<0.001
No	171	51.8	3461	84.3	573	90.2	4205	90.2	
**Drunk alcohol in the first three months of pregnancy**	** **	** **	** **	** **	** **	** **	** **	** **	
Yes	102	58.3	574	47.8	119	52.2	795	49.6	0.59
No	73	41.7	621	51.7	109	47.8	803	50.1	
**Drunk alcohol since the fourth month of pregnancy**	** **	** **	** **	** **	** **	** **	** **	** **	
Yes	43	24.4	459	38.4	105	47.1	607	38.1	<0.001
No	133	75.6	733	61.4	118	52.9	984	61.8	
**Used recreational drugs during pregnancy**	** **	** **	** **	** **	** **	** **	** **	** **	
Yes	18	5.5	42	1.0	1	0.2	61	1.2	<0.001
No	312	94.5	4062	99.0	632	99.8	5006	98.8	
**BMI Category**	** **	** **	** **	** **	** **	** **	** **	** **	
Underweight (Below 18.5)	23	7.1	183	4.6	5	0.8	211	4.3	<0.001
Healthy weight (18.5–24.9)	200	61.7	1837	46.4	200	32.8	2237	45.7	
Overweight (25–29.9)	63	19.4	1142	28.8	224	36.8	1429	29.2	
Obese (30 or higher)	38	11.7	798	20.2	180	29.6	1016	20.8	
	**N**	**Mean** **(95% CI)**	**N**	**Mean** **(95% CI)**	**N**	**Mean** **(95% CI)**	**N**	**Mean** **(95% CI)**	**p =**
**BMI at booking appointment**	324	23.7 (23.2 to 24.3)	3972	25.9 (25.7 to 26.1)	609	28.0 (27.5 to 28.4)	4905	26.0 (25.9 to 26.2)	<0.001
**IMD Score**	330	45.1 (43.1 to 47.0)	4117	43.0 (42.5 to 43.6)	635	37.3 (35.8 to 38.7)	5082	42.4 (42.0 to 42.9)	<0.001
**Number of weeks gestation at booking appointment**	311	13.9 (13.5 to 14.4)	3905	12.7 (12.6 to 12.8)	602	13.1 (12.8 to 13.3)	4818	12.8 (12.7 to 12.9)	<0.001

*General Certificate of Secondary Education—subject specific examinations taken at the end of compulsory education at age 16 in England

** Advanced Level—non-compulsory subject specific examinations taken at age 18 in England

### Nutrition related variables

Analysis of the nutrition related variables (Table 2) showed that young women aged 19 and under were less likely to have used any vitamins or iron supplements in the 4 weeks preceding completing the questionnaire compared to other age groups. Adolescents were also the least likely group to have used Pregnacare multi-vitamins at least twice per week during their pregnancy. While there was no differences between age groups in awareness of the recommendations to eat at least five portions of fruit and vegetables a day the adolescent group were less likely than older women to achieve this recommendation. The adolescent group consumed significantly more sugar sweetened cola compared to older women; there were no statistically significant differences in consumption of artificially sweetened cola between age groups.

**Table 2:** Maternal age and nutrition related variables.

	≤19		20–34		35≥		Total		
	N	%	N	%	N	%	N	%	p =
**Used any vitamins or iron supplements in the last 4 weeks**	** **	** **	** **	** **	** **	** **	** **	** **	
Yes	86	26.1	1606	39.1	318	50.1	2010	39.6	<0.001
No	244	73.9	2500	60.9	317	49.9	3061	60.4	
**Used Pregnacare multivitamins at least twice per week**									
Yes	21	6.4	553	13.4	114	18.0	688	13.5	<0.001
No	309	93.6	3565	86.6	521	82.0	4395	86.5	
**Are you familiar with the 5 a day recommendations for fruit and vegetables?**									
Yes	303	95.6	3279	95.5	500	97.1	4082	95.7	0.238
No	14	4.4	156	4.5	15	2.9	185	4.3	
**Do you consume 5 portions of fruit and vegetables per day?**									
Always	28	8.8	586	17.0	126	24.5	740	17.3	<0.001
Sometimes	259	81.7	2616	76.0	365	70.9	3240	75.8	
Never	30	9.5	238	6.9	24	4.7	292	6.8	
**Where does most of your advice about healthy eating during pregnancy come from?**									
Family members	124	37.6	845	20.5	83	13.1	1052	20.7	<0.001
Midwives/Health visitors	119	36.1	1175	28.5	155	24.4	1449	28.5	
Magazines/Newspapers	31	9.4	490	11.9	99	15.6	620	12.2	
GPs/Doctors	16	4.8	126	3.1	13	2.0	155	3.0	
Books	11	3.3	395	9.6	85	13.4	491	9.7	
Other	11	3.3	310	7.5	66	10.4	387	7.6	
Friends	5	1.5	96	2.3	13	2.0	114	2.2	
	**N**	**Mean** **(95% CI)**	**N**	**Mean** **(95% CI)**	**N**	**Mean** **(95% CI)**	**N**	**Mean** **(95% CI)**	**p =**
Cups of regular (sugar sweetened) cola per day	330	0.9 (0.7 to 1.1)	4269	0.4 (0.4 to 0.4)	484	0.2 (0.1 to 0.2)	5083	0.4 (0.4 to 0.4)	<0.001
Cups of diet (artificially sweetened) cola per day	330	0.2 (0.1 to 0.2)	4269	0.1 (0.1 to 0.1)	484	0.1 (0.1 to 0.2)	5083	0.1 (0.1 to 0.1)	0.674

Adjustment for ethnicity and IMD score in the regression analysis produced similar results as shown in Table 3.

**Table 3:** Dietary behaviour by maternal age.

	Crude OR (95% CI)		aOR (95% CI)*	Crude OR (95% CI)		aOR (95% CI)*
	≤19 Years (n = 641)^†^			35≥ Years (n = 1427) ^†^		
**Used any vitamins or iron supplements in the last 4 weeks**						
Yes	0.54 (0.42 to 0.70)	0.71 (0.55 to 0.92)		1.53 (1.27 to 1.85)	1.56 (1.29 to 1.89)	
No	Ref	Ref		Ref	Ref	
**Used Pregnacare multivitamins at least twice per week**						
Yes	0.44 (0.28 to 0.68)	0.44 (0.28 to 0.70)		1.49 (1.16 to 1.90)	1.33 (1.04 to 1.71)	
No	Ref	Ref		Ref	Ref	
**Familiar with the 5 a day recommendations for fruit and vegetables**						
Yes	1.01 (0.58 to 1.77)	0.62 (0.35 to 1.12)		1.46 (0.80 to 2.65)	1.26 (0.67 to 2.24)	
No	Ref	Ref		Ref	Ref	
**Consume 5 portions of fruit and vegetables per day**						
Always	0.37 (0.22 to 0.63)	0.35 (0.20 to 0.61)		1.96 (1.18 to 3.23)	1.68 (1.01 to 2.80)	
Sometimes	0.77 (0.52 to 1.15)	0.68 (0.45 to 1.03)		1.20 (1.18 to 3.23)	1.11 (0.69 to 1.78)	
Never	Ref	Ref		Ref	Ref	
**Where does most of your advice about healthy eating during pregnancy come from?**						
Family members	4.26 (2.27 to 8.00)	5.65 (3.14 to 10.18)		0.49 (0.33 to 0.72)	0.56 (0.36 to 0.88)	
Midwives/Health visitors	2.94 (1.56 to 5.51)	2.77 (1.54 to 4.99)		0.69 (0.49 to 0.99)	0.78 (0.53 to 1.16)	
Magazines/Newspapers	1.76 (0.87 to 3.55)	1.70 (0.86 to 3.36)		0.84 (0.57 to 1.25)	0.83 (0.53 to 1.29)	
GPs/Doctors	3.66 (1.65 to 8.10)	3.92 (1.86 to 8.25)		0.46 (0.22 to 0.97)	0.62 (0.31 to 1.24)	
Books	0.79 (0.34 to 1.84)	1.09 (0.51 to 2.35)		1.05 (0.70 to 1.56)	1.03 (0.66 to 1.61)	
Friends	1.49 (0.50 to 4.38)	1.61 (0.64 to 4.05)		0.60 (0.29 to 1.27)	0.71 (0.36 to 1.42)	
Other	Ref	Ref		Ref	Ref	
	Unadjusted β (95%CI)	Adjusted β (95%CI)*		Unadjusted β (95%CI)	Adjusted β (95%CI)*	
Cups of regular (sugar sweetened) cola per day	0.49 (0.36 to 0.62)	0.46 (0.33 to 0.59)		-0.22 (-0.33 to -0.12)	-0.20 (-0.31 to -0.09)	
Cups of diet (artificially sweetened) cola per day	0.03 (-0.04 to 0.09)	-0.02 (-0.09 to 0.05)		0.01 (-0.05 to 0.07)	0.94 (-0.06 to 0.05)	

*Adjusted for index of multiple deprivation score and maternal ethnicity

†comparison to reference category women aged 20–34 years

### Results of the principal component analysis

Several combinations of food groupings were explored to see which gave the most sensitive output in the PCA. These included loading all of the food items in separately, separating whole grains from other grains and starches, analysing all proteins as one group and separating proteins by type (red meat, poultry and fish). The groupings outlined in Table 4 explained the highest amount of variance; therefore these were the input variables which were selected. Loading all of the foods individually returned very low levels of correlation between the variables, meaning that a very large number of factors (11) were returned. This suggested that grouping the variables to form new variables would improve the correlation of intake between food types and therefore make the PCA more meaningful.

**Table 4:** Food groupings for principal components analysis.

Potatoes	Grains and Starches	Whole meat and fish	Meat in Sauce	Processed meat and fish	Cured Pork Products	Savoury Snacks	Sweet Snacks
Chips	Fibre or bran-rich wheat breakfast cereal	Beef	Chicken or turkey in sauce	Beef burgers	Bacon	Potato Crisps	Cakes, buns, gateaux, doughnuts, muffins
Roast or fried potatoes	Oat cereals	Lamb	Beef, lamb or goat in sauce	Kebabs	Ham	Other salted savoury snacks	Sweet pastries
	Crispbread	Pork	Pork in sauce	Meat pies and pastries	Cured sausage		Chocolate bars and chocolate coated biscuits
	Other breakfast cereals	Chicken or turkey	Gravy made with pan or meat juices (not instant)	Sausages			Sweet biscuits
	Pasta or noodles	White fish		Hotdogs			
	Savouries like Yorkshire pudding, pakoras etc.	Tinned tuna		Chicken or turkey nuggets			
		Fresh or tinned oily fish		White fish in batter or breadcrumbs			
		Smoked fish					
		Salted or dried fish					

When the analysis was completed without stipulating the number of factors to be retained two factors were retained which explained 43.8% of the total variance. On closer examination of the output, including examination of the inflection point of the scree plot, it was considered that a third and fourth factor with Eigen values of 0.971 and 0.939 respectively should be considered for inclusion. The analysis was therefore run with both 3 and 4 factors stipulated and the results compared with the initial analysis. The inclusion of a third factor presented a distinct dietary pattern which added to the logical understanding and interpretation of the data. The inclusion of a fourth factor did not however add to the understanding of the data. The decision was therefore made to retain 3 factors from the analysis, the inclusion of these 3 factors would then account for 55.9% of the total variance. Details of the retained factors and factor loadings are given in Table 5, factor loadings were considered to be very good where they were 0.6 or above and moderate where they were 0.45–0.59[13]. The PCA therefore identified three independent variables whose relationship to other variables in the data set could be considered.

**Table 5:** Dietary patterns identified by principal component analysis.

	Component		
	1 Snack and processed foods	2 Meat and fish	3 Grains and starches
Savoury Snacks	0.756		
Potatoes	0.691		
Sweet Snacks	0.666		
Processed meat and fish	0.510	0.480	
Whole meat and fish		0.794	
Meat in Sauce		0.729	
Cured pork products		0.470	
Grains and Starches			0.888

### Association between dietary pattern and maternal age

The analysis showed that there was an association between both the snacks and processed foods pattern and the grains and starches pattern and maternal age group (Table 6). The mean PCA score for snack and processed foods was highest in the adolescent group. The opposite was observed in the case of the grains and starches dietary pattern where women aged 35 and over had the highest mean PCA score. There were no statistically significant differences between groups relating to the meat and fish dietary pattern. The results of these analyses are shown in Table 6.

**Table 6:** Differences in dietary pattern scores^†^ by maternal age.

	≤19		20–34		35≥		Total		
	N	Mean PCA Score (95% CI)	N	Mean PCA Score (95% CI)	N	Mean PCA Score (95% CI)	N	Mean PCA Score (95% CI)	p =
Snack and Processed Foods	277	0.6 (0.4 to 0.8)	2949	-0.02 (-0.1 to 0.01)	320	-0.3 (-0.4 to-0.2)	3546	0.0 (-0.03 to 0.03)	<0.001
Meat and Fish	277	0.04 (-0.1 to 0.2)	2949	-0.01 (-0.04 to 0.03)	320	0.04 (-0.05 to 0.1)	3546	0.0 (-0.03 to 0.03)	0.55
Grains and Starches	277	-0.04 (-0.2 to 0.1)	2949	-0.01 (-0.05 to 0.02)	320	0.1 (0.03 to 0.3)	3546	0.0 (-0.03 to 0.03)	0.03

† Dietary pattern scores derived from Principal Component Analysis indicating the extent to which participants adhere to the dietary pattern

## Discussion

The results show an association between two of the dietary patterns in this analysis and maternal age. Strongest associations were seen in the snack and processed foods pattern where women aged ≤19 were found to have the highest mean PCA score reflecting a higher intake of foods that are typically viewed as snack items (including crisps, chocolate, biscuits and cake) and moderate intake of processed meat. Adolescent women also reported consuming higher levels of sugar sweetened cola compared to older women; no differences were observed in levels of consumption of artificially sweetened cola.

There were no differences by age group in women's awareness of recommendations to eat five portions of fruit and vegetables per day; however adolescent women were less likely to say they achieve this recommendation. Adolescent women in the sample also reported the lowest use of nutritional supplements.

Adolescent women had a higher prevalence of underweight (BMI <18.5) and older women a higher prevalence of overweight or obesity (BMI >25).

These results are largely consistent with findings from the latest wave of the National Diet and Nutrition Survey (NDNS)[14]. Results from the 2012/13–2013/14 surveys found significantly fewer adolescent girls reported meeting 5-a-day recommendations and had higher intakes of sugar sweetened beverages compared to adult women. The prevalence of overweight and obesity in the NDNS was also in line with this cohort (58% among adult women and 38% among girls aged 11–19). Whilst the observation that a population with an overall poorer quality diet would have lower levels of overweight and obesity is counterintuitive, however the impact of poor diet on BMI status is cumulative meaning that the effects of poor diet on the adolescent population will be evident in time if dietary changes are not made[15]. These similarities suggest that the findings of the current study are likely to be reflective of the UK population. Dietary patterns during pregnancy have been previously examined using similar methods. One UK study[16] found an association between dietary pattern and maternal age with the 'health conscious' pattern being associated with increasing age and the opposite being true of the 'processed' pattern identified in the study. A further study conducted in New Zealand[17] similarly found a positive association between increasing maternal age and 'health conscious' and 'fusion/protein' dietary patterns and the opposite association with 'junk' and 'traditional/white bread' patterns.

The findings of these studies are consistent with those of the present study however neither specifically addressed adolescent women or older women as distinct groups. As discussed in the introduction, the nutritional needs of adolescent women in particular may differ from those of older women due to the continued growth of the mother meaning that understanding the nutritional intake of young women during pregnancy is important. This study helps us to start to understand the dietary patterns associated with this group, however further work based on more robust dietary surveys is needed in order to improve understanding of this complex topic. A particular strength of this work is that it utilises well-established, ethnically diverse, UK based cohort data in a way which is unique to this study. This cohort includes a large number of participants meaning that analysis by maternal age including an adolescent group is both possible and robust.

There were limitations in the dietary data available for this study. The number of items included in the FFQ was limited meaning that there is potential for the dietary patterns identified to be lacking in detail, or that additional patterns which might be present in the population could have been missed. This issue also resulted in the distribution of dietary pattern scores being significantly skewed, meaning that it was not possible to use statistical modelling to predict the extent to which maternal age influences dietary pattern, only that there appears to be an association. There was also no definition of portion size in the collection of data meaning there is likely to be significant variation between participants self-defined portion sizes. The FFQ was completed at 26–28 weeks of pregnancy and related to the preceding 4 weeks, meaning that the dietary patterns identified are only reliably relevant to the second trimester of pregnancy. Women's diet may change over the course of pregnancy[18] meaning that these results may not be generalisable to the first and third trimesters.

This study assesses dietary patterns ascertained by principal component analysis. Dietary patterns have been defined as "foods that are actually consumed in various characteristic combinations"[19]. The study of dietary patterns as opposed to single foods or nutrients has the advantages of allowing for interactions between nutrients to be accounted for and reflecting more accurately that people do not eat single foods or nutrients, they eat meals comprising of many food types. Dietary patterns can be derived either theoretically as in the case of the Diet Quality Index[20] where foods are ranked based on what current knowledge defines as healthy or less healthy or empirically using statistical methods to reduce collected dietary data into distinct patterns. A systematic review of the use of these methods in nutritional epidemiology has concluded that empirically derived eating patterns may improve our understanding of eating behaviour and therefore provide a stronger evidence base from which to provide dietary advice[21]. For these reasons this method of assessing dietary patterns is considered to be appropriate for this study.

The results suggest that there may be some cause for concern regarding the quality of adolescent pregnant women's diet, despite reporting similar levels of knowledge of recommendations to older women. This suggests that factors other than knowledge may be important in developing behaviour change interventions. There are a number of different theoretical models discussing the process of translating knowledge into action in public health. For example the health belief model[22] is based on the premise that individuals will make an assessment as to whether the benefits of a change in behaviour outweigh the perceived costs. This includes an assessment of both the risks associated with not changing behaviour, their belief in the potential benefits and the perceived barriers to taking action. Applied to this data therefore there are several avenues for further investigation with the aim of encouraging positive dietary changes.

The adolescent women in the sample also reported less use of nutritional supplements compared to the reference group. This suggests that any gaps in the nutritional profile of young women during pregnancy as a result of a sub-optimal diet may not be being filled by supplementary vitamins and minerals and may therefore leave women in this group with nutrient deficiencies.

Consumption of sugar and artificially sweetened cola in this cohort has previously been examined[23] with results showing that high intakes of sugar sweetened cola were associated with higher odds of preterm delivery in the cohort as a whole. This suggests sugar sweetened cola intake in adolescent women may be a cause for concern.

Energy intake was not available for analysis in this dataset meaning that maternal BMI at booking appointment is a useful indicator. Individual differences in total energy intake are largely determined by a combination of body size, physical activity and metabolic efficiency. Measurements of energy intake and physical activity in epidemiology are often crude and suffer from under or over reporting, and metabolic efficiency is essentially impossible to measure in this setting. For these reasons height and weight measurements are often considered a suitable alternative to measures of energy intake[24]. The results of this study suggest that for some adolescent women their overall energy intake during pregnancy may be insufficient while an association with overweight and obesity in older women suggests an excessive energy intake.

The results of this study are consistent with previous work assessing maternal obesity in the UK[25]. This work found that levels of maternal obesity (measured during the first trimester) increased as maternal age increased. Increases in maternal obesity impact significantly on the health of mothers and babies and increase the need for specialist and high dependency care, therefore having both health and economic implications.

## Conclusions

There may be some cause for concern with regard to diet quality in adolescent women and obesity among older women during pregnancy. Further work using more comprehensive methods of dietary data collection would be advantageous in assessing the nature of these relationships.

The impact of dietary pattern on birth outcomes is also an important area for further work, particularly in light of evidence presented previously that there is a higher risk of extremely low birthweight babies and extremely pre-term delivery among adolescent women in this cohort[26].

Interventions targeted to specific age groups to facilitate behaviour change may be useful due to differences identified in the types of nutritional issues affecting pregnant women at different ages. There is a need to further understand the barriers to healthy eating during pregnancy in order to develop successful interventions.
